# Healthcare professionals’ perceptions of participation and learning related to mealtimes in long-term care facilities: an interview study

**DOI:** 10.1186/s12877-026-08358-x

**Published:** 2026-09-24

**Authors:** Camilla Allert, Stefan Andersson, Nina Carlsson, Anna Sandgren

**Affiliations:** 1 Present Address: Department of Health and Caring Sciences, Linnaeus University, Kalmar/Växjö, Sweden; 2 Center for Collaborative Palliative Care, Linnaeus University, Växjö, Sweden

**Keywords:** Healthcare professionals, Learning, Long-term care facilities, Older adults, Participation, Person-centredness, Reflexive thematic analysis

## Abstract

**Background:**

Mealtimes in long-term care facilities are complex and serve multiple purposes. They provide opportunities for participation and learning, involving residents, relatives and healthcare professionals, each with unique circumstances. Participation and learning are central to healthy ageing. Healthcare professionals play a key role in enabling participation and learning among those involved, although these opportunities may be shaped by their perceptions and attitudes. Therefore, this study aimed to explore healthcare professionals’ perceptions of participation and learning among residents, their relatives, and colleagues related to mealtime situations in long-term care facilities.

**Methods:**

Data were collected through individual semi-structured interviews with 23 healthcare professionals (nursing assistants, licensed practical nurses, and registered nurses) working in long-term care facilities in a municipality in south-eastern Sweden. The data were analysed using reflexive thematic analysis.

**Results:**

Three themes were generated: (1) *Shaped by relationships and circumstances*, (2) *Rewarding yet challenging*, and (3) *Prerequisites influence opportunities.* Participation and learning related to mealtime situations manifested in various ways and to differing degrees, from extensive to almost non-existent. It was a balancing act for the healthcare professionals managing multiple challenges to enable participating and learning; however, residents and relatives were seldom invited to engage in this problem-solving. For participation and learning to occur, prerequisites were required at all levels in the organisation.

**Conclusion:**

Healthcare professionals perceive that participation and learning among residents, relatives, and colleagues in long-term care facilities do occur, but to varying degrees and are associated with multiple challenges. Drawing on a learning model, such as Illeris, can help identify which components need to be developed, which together with a deeper understanding and implementation of person-centredness, could contribute to increased participation and learning, and thereby to the wellbeing of residents, relatives, and healthcare professionals. However, this approach must permeate the entire organisation.

**Supplementary Information:**

The online version contains supplementary material available at https://doi.org/10.1186/s12877-026-08358-x.

## Background

Learning and participation, such as decision-making, are two important areas of ability needed to live a life one values, to enable healthy ageing and well-being in older age. These abilities must be supported by environments that help maintain them and are based on a person’s physical and mental capacities [1]. Participation is emphasized by the World Health Organization [WHO] and can be understood as the opportunity to be involved in one’s own life situation [2]. This includes e.g. being listened to, having one’s views considered important, and having the right to make decisions about oneself [3]. Participation is also a legal right, highlighted in several Swedish laws, including the Social Service Act [4] and the Patient Act [5]. Ageing involves physiological changes that can affect both physical and cognitive capacity and increased risk of various diseases [6], and multimorbidity may lead to challenges in maintaining independence and performing daily activities [7]; for those requiring extensive support, long-term care facilities may represent an appropriate alternative.

In long-term care facilities, mealtime situations constitute a central and complex part of daily life [8–11]. A systematic review by Watkins et al. [11] found that staff perceived mealtimes as significant for residents’ quality of life, with several influencing aspects confirmed by later research. Social interactions with residents, relatives, and healthcare professionals were highly valued but could be affected by residents’ diseases and functional abilities [8–12]. Nutrition and the way it was served were also central, including adapting food texture and portion size [8–12]. Additionally, the environment, such as the dining area, should be designed to meet residents’ needs, including appropriate cutlery and tableware [8–9, 11]. However, mealtimes could also be challenging when the behaviour of other residents or interpersonal conflicts affected the atmosphere [8, 10–12], leading to distractions among residents [13]. In addition, feelings of shame related to eating performance could also discourage residents from participating in shared mealtimes [8, 12]. Mealtimes could also place pressure on the staff to be able to meet all demands [8, 11, 13].

Multiple interventions have been implemented to improve mealtimes in long-term care facilities. In a review by Heikkilä et al. [14], interventions focused on aspects of the environment, social interactions, food, organisational systems (e.g. leadership and economy), and the overall atmosphere, while Passos et al.’s [15] review focused on interventions to address mealtime support. One area identified was training programs for healthcare professionals, with feeding skills being the most common focus, consistent with the findings of Faraday et al.’s [16] review. However, they also found that training programs included the environment, nutritional knowledge, and social aspects, and the program content appeared to address the identified educational needs related to mealtimes. Healthcare professionals were motivated to participate [15–17], expressed a desire for continuous education [9, 18], and had more positive attitudes after completing nutrition-related training [19, 20]. Some learning programs had explicit educational foundations. Faraday et al.’s program [21] was grounded in health education and learning principles, including collaboration, applicability, interactivity and the inclusion of the residents’ perspectives. Similarly, the model developed by Keller et al. [22] was grounded in relationship-centred principles, emphasising support, dignity, enjoyment, and engagement with residents’ opportunities. Several studies mentioned the importance of the team and its ability to work collaboratively [18, 20–22], however, the opportunity and value of learning from one another were described only by Passos et al. [18] and Faraday et al. [21]. Although aspects and principles of participation and learning were included in mealtime interventions, healthcare professionals’ perspectives on these concepts remain unclear.

Participation and learning are closely linked to person-centred care. Previous research [23–26] and both international [1] and national guidelines emphasise that elderly care, including mealtimes in long-term care facilities, should be person-centred [27]. According to the Person-centred Practice Framework [28], one goal is a healthful culture, a setting that enables human flourishing, a possibility to grow and to push one’s boundaries, while also achieving stillness and harmony. To reach this goal, the whole organisation is important, from policies and leadership to the staffs’ abilities and beliefs [28]. Participation is a key component in person-centred practice and may be expressed through shared decision-making, enabling persons to engage in decisions and have their values and concerns considered [28]. A metasynthesis by Vaismoradi et al. [29], describes that older adults in elderly care generally experience the care as person-centred and view participation as a fundamental right, expecting their wishes to be taken into consideration. When these expectations are not met, dissatisfaction may arise, which can lead to residents choosing to be even less involved. A lack of autonomy has also been reported [30], which can reduce the opportunities for participation [31, 32].

However, Mortensen et al. [33] found that some degree of paternalism may be acceptable, if the resident has been asked about their opinion beforehand. Yet, to integrate person-centred care, a holistic perspective is essential, involving not only older adults and professionals across health and social care sectors, but also relatives [34]. Previous research has shown that relatives experience shortcomings in their participation in elderly care. They express a desire to be involved in decision-making regarding their next of kin, and feel that their knowledge is often underutilised [35–37]. They want to participate by communicating their concerns, but they do not always do so, as they fear that staff may perceive it as criticism [36]. Staff perceptions of relatives have shown that they can be viewed as a resource, someone to obtain information from, to certain extent, but they are also perceived as demanding, time-consuming, and troublemakers [38, 39]. However, participation may also provide opportunities for learning.

Learning can be defined in different ways; one way is as “any process that in living organism leads to permanent capacity change and which is not solely due to biological maturation or ageing” [40, p. 3]. This broad definition indicates that learning can occur among relatives and older adults, as well as healthcare professionals. Thus, learning can be understood as acquiring new knowledge, such as when healthcare professionals learn theoretical or practical knowledge. However, learning can also be understood as older adults training to preserve their abilities to counteract the capacity changes that diseases may otherwise cause. According to Illeris [41], learning can occur both consciously and unconsciously. To broaden and deepen understanding of the complex processes of learning in mealtimes situations in long term-care facilities, Illeris’ learning model [41] provides a useful framework. The model comprises three dimensions and two processes, all of which are often closely linked. The dimensions are content, incentive, and interaction, all of equal importance to enabling learning. The content dimension comprises knowledge, skills, and understanding; the incentive dimension consists of emotions, motivation, and volition; while the interaction dimension concerns communication, cooperation, and action. The dimensions can be viewed as the components involved in learning, while the processes of learning describe how it happens. The interaction process is an external process between the learner and the environment, which may be material (e.g. learning materials, workplace), social (e.g. colleagues, organisation), and cultural (e.g. social norms, educational traditions). In contrast, acquisition is an internal process that takes place within the learner. Participation can be conceptualized as both part of the interaction process and the interaction dimension [41]. However, interpretations of participation vary, and based of the definition presented above, social interaction does not necessarily constitute participation. Therefore, while participation and learning are often closely associated, participation cannot be assumed to be a prerequisite for learning.

Participation and learning are important for promoting healthy ageing and wellbeing [1]. However, residents in long-term care facilities often have multiple diseases and complex needs [6, 7], making them dependent on staff working in these facilities. At the same time, relatives remain important sources of support and significant people in residents´ lives, whose knowledge and contributions are valuable. Healthcare professionals are likely to interact with all of these perspectives, making their perceptions important. Previous research has examined healthcare professionals’ perceptions of mealtimes in long-term care facilities and the implementation of interventions to address identified knowledge needs. Although some interventions have incorporated learning principles, research on staff perceptions of participation and learning among those involved in mealtime situations remains limited. Therefore, this study aimed to explore healthcare professionals’ perceptions of participation and learning related to mealtime situations among residents, their relatives, and colleagues in long-term care facilities.

## Methods

### Design

To reach a deeper understanding of the participants’ perceptions of the two phenomena of participation and learning, an inductive explorative design was chosen. Ontologically and epistemologically, the study stands on a social constructivist ground, where the meaning is subjective and related to the context, but also created in interaction with others [42]. The importance and acknowledgment of the researchers’ subjectivity and reflexivity are coherent with the chosen reflexive thematic analysis [43]. The study is reported according to the Consolidated Criteria for Reporting Qualitative Research checklist [44], (Additional file 1), and guided by the Reflexive Thematic Analysis Guidelines [45].

### Setting

The study was conducted at long-term care facilities in a medium-sized municipality in southern Sweden. The median age for moving to a long-term care facility in Sweden is 86.1 years, and the median length of stay is 27 months. Often, residents have several diseases or impairments, with complex healthcare needs [46]. Legislation and governing documents regarding Swedish elderly care emphasize the independence of the older adults [4] and the importance of using life story documents as a foundation for person-centred care. Relatives can play an important role in the development of life stories [47]. The importance of including the perspectives of relatives and enabling their participation is also emphasized [48]. In this study, four publicly funded long-term care facilities were included, each consisting of three to eight units, with seven-16 residents living in private apartments within each unit. Three of the four facilities had separate dementia units, although residents with cognitive impairments could also reside on general care (somatic) units. Each unit had a dining room that served as the primary location for meals. Three main meals were served: breakfast, lunch, and dinner. Lunch and dinner were provided at predetermined times, while most residents could choose when to have breakfast. The food was prepared in central kitchens, either located within the included facilities, or transported from a nearby site. All facilities offered three meal options for lunch and two for dinner, including a vegetarian option. Breakfasts included a variety of options, and during the other meals, accompaniments were available for residents to choose from. While some units still used trays to serve food, most served the food on a bench, a serving cart, or bowls placed on a table. All facilities embraced the concept of social pedagogical meals, which allowed staff to take a small portion of food and dine alongside residents.

A meal concept had been implemented in the long-term care facilities to varying degrees, with the aim of increasing resident participation and creating a more pleasant mealtime experience. The concept included opportunities for residents to choose their main meals, preparing the dining room in an appealing manner, ensuring sufficient time for meals, enabling residents to serve themselves, and facilitating social participation during mealtimes.

At the units, daily care was provided by licensed practical nurses or nurse assistants. Among these staff members, at least one per unit had the role of meal representative, with special responsibility for mealtime situations, this role is usually held by licensed practical nurses. In the municipality, collaboration shifts were used, meaning that licensed practical nurses and nurse assistants had some shifts each month at other long-term care facilities within the municipality where there was a shortage of personnel. Registered nurses were based at the long-term care facilities, but they did not participate in the daily care on the units. Instead, they functioned more as consultants who came when needed. They were responsible for supervising and educating licensed practical nurses and nurse assistants in care related to mealtimes and nutrition, and also maintained contact with relatives, providing and receiving information about residents.

### Recruitment and participants

Participants were recruited using a purposive sample strategy [49]. The inclusion criteria were: nurse assistants, licensed practical nurse, specialist licensed practical nurse or registered nurse connected to the long-term care facility. The managers informed the coordinator at long-term care facilities to schedule interview times during working hours according to the inclusion criteria and for nurse assistants and licensed practical nurses they were told to schedule those who were currently or previously meal representatives. The managers were not involved in selecting potential participants. The coordinator identified individuals who were available during the interview period and forwarded an information letter about the study to 26 potential participants. In total, 23 participants confirmed their appointment and took part in individual interviews: 15 licensed practice nurses, one nurse assistant, and seven registered nurses. Three potential participants did not accept their appointment and no reason was given. Characteristics of the participants are available in Table 1.

**Table 1:** Characteristics of participants

	All *N* = 23
Age, Mn [min-max]	45 [25–65]
Gender, *n* (%)	
Woman	21 (91)
Men	2 (9)
Profession, *n* (%)	
Nurse Assistant	1 (4.3)
Licenced practical nurse	15 (65.2)
Registered nurse	7 (30.5)
Time worked in healthcare (years), Mn [min-max]	20 [1–47]
Time worked in elderly care (years), Mn [min-max]	11 [1–47]
Main employment in dementia ward, *n* (%)	
Yes	6 (26)
No	17 (74)

### Data collection

Data were collected through individual semi-structured interviews. All interviews were conducted at the long-term care facilities, in a quiet and secluded room. The interviews were guided by the overarching questions: “If you imagine a typical mealtime situation for the residents at your workplace, who participates in it?”; “In what way are they allowed to participate?”; and “When people participate, it can also mean an opportunity to get new ideas and learn new things. How do you see the opportunity to learn from and with colleagues, residents, and relatives?”. To encourage narration and to check and deepen understanding, probing questions such as “Can you explain?”, “Can you give an example?”, “How do you handle suggestions?”, and “In what ways could you learn?” were asked. An English translation of the interview guide is available in Supplementary file 1. After the first interview, the authors discussed the outcome to assess whether the questions elicited data relevant to the study aim; no changes were made. The interviews were performed by the first author, between March and May 2025. They lasted 27 to 92 min (median time 50 min) and provided sufficient depth and richness to address the research aim. All interviews were audio-recorded and transcribed verbatim by the first author, and names of people, long-term care facilities or places were pseudonymised.

### Data analysis

The data analysis was based on the six phases in the guidelines for reflexive thematic analysis as described by Braun and Clarke [43]. The process was circular; a return to the different phases was performed continuously in the analysis. The analysis started during the transcription, which involved repeated listening to the interviews, leading to familiarising with data. The immersion continued by repeated reading of the transcripts but also through a critical engagement with the data, an alternation between proximity and distance, in an attempt to see the meaning. In the coding phase, segment in data which met the aim of the study were market and given a code label, first at a more descriptive level and further on, at a more interpretative level. The initial coding was performed by the first author. Through collaboration with the co-authors, alternative interpretations were explored, resulting in richer insights and a deeper understanding of the data. After several coding rounds, when code labels alone captured the data and its diverse meanings, the analysis proceeded to generating initial themes. The first candidate themes were written in a word processing software but in the continuing process data were collated, codes were printed, clustered, and re-clustered manually to each candidate theme. The themes were then developed and reviewed by re-reading all codes and the entire data set to determine whether the candidate themes captured the most important patterns from the interviews, and whether each theme had a distinct focus. Some initial themes were discarded, and new ones created. In the next phase, mind-maps of the content were drawn, overlapping areas were identified, and themes were refined and named. The theme structure (code labels, themes, and subthemes) is available in Additional file 2. The writing process was ongoing throughout the analysis, and in the final phase, details were shaped and interview quotes were added to illustrate the results. During the whole process, the first author kept a reflexive journal, and all authors discussed the meaning of the content using the researchers’ subjectivity as a resource.

### Ethical considerations

The study was approved by the Swedish Ethical Review Authority (Ref. no: 2025-04620-01). Before the interviews began, participants again received written and oral information about the study and were given the opportunity to ask questions before signing the informed consent form. The participants were also informed that they could withdraw from the study at any time without providing a reason for their withdrawal. The interviews were conducted by the first author, who had no prior relationship with the participants, in their workplace. Conducting the interviews in this setting may have limited their willingness to speak openly. However, the interviews took place in a quiet, secluded room, and efforts were made to create a relationship and a safe atmosphere, which may have helped reduce the power imbalances [50]. The fact that several interviews were relatively lengthy may indicate that the participants felt comfortable sharing their perceptions. The study was conducted in compliance with the ethical guidelines of the Declaration of Helsinki [51].

## Results

Healthcare professionals’ perceptions of participation and learning related to mealtime situations among residents, relatives, and colleagues in long-term care facilities were captured in three themes generated from the data: *Shaped by relationships and circumstances*; *Rewarding yet challenging*; and *Prerequisites influence opportunities*.

### Shaped by relationships and circumstances

Participation and learning manifested in various ways among residents, their relatives, and healthcare professionals; however, participation was more common. Even though healthcare professionals perceived mutual learning as something natural, its expressions were not always obvious, and when expressed, it was often in a more general way. Most healthcare professionals attempted to enable residents to participate to the greatest extent possible, which contributed to learning by preserving their abilities. At the same time, they were aware that some colleagues did not involve residents, resulting in considerable variation in the level of participation and learning, from extensive to almost non-existent, depending on who was on duty.

Healthcare professionals perceived that residents’ participation in relation to the mealtime situations could be expressed in several ways. These included the opportunity to select meals, serve themselves, pass bowls to one another or assist each other, engage in conversation during meals and remain at the dining table, even after finishing their food. For residents who chose to eat in their apartment, healthcare professionals enabled participating and learning by sitting with them, letting them serve themselves and sharing a social pedagogical meal. They found ways to support participation and learning among residents with cognitive and physical impairments by helping them choose and marking the chosen dish, and allowing them to select meals even if they might later forget what they had ordered. For residents unable to serve themselves, healthcare professionals promoted participation by allowing them to see the food and observe as it was placed on their plates. Even when healthcare professionals were familiar with residents’ usual preferences, they continued to support participating by asking what and how much they preferred.

“He who always takes fish, you always ask anyway,… I always use to say: I know your answer, but do you want this or that?… he always wants it (fish), but you ask him anyway. Suddenly, he changes, you do not know that.” [Participant 6].

Learning from residents’ included learning about their past experiences with food, meal preparation practices, preferences, and cultural backgrounds. It also occurred when healthcare professionals observed what residents appeared to dislike or had difficulty eating, and communicated this to the central kitchen to suggest adjustments. However, there were a lack of discussion with residents, as healthcare professionals perceived that residents were generally satisfied with the food, which limited opportunities for mutual learning. Similarly, mutual learning did not always occur with relatives, as some healthcare professionals did not perceive it their responsibility to explain why a different approach was required. When explanations were provided, however, shifts in relatives’ attitudes and understanding were perceived. Participation and learning among relatives were also enabled when healthcare professionals requested information about the residents.

Mutual learning were common both among colleagues and across professions; however, it was not always linked to the mealtime situation but rather related to the work in general. It was considered natural to ask a colleague when encountering something one had not yet learned of or had no previous experience with, thereby making use of each other’s different abilities.

“I usually think that no one will know everything, so it is very nice to be able to take advantage of each other’s knowledge. Someone may be great on computers, someone may be the one successfully bringing everyone in the shower, someone may be great at structure. There is always someone who is better at something. Dare to ask, and then learn.” [Participant 5].

To determine what worked best for individual residents, or for the group, different approaches were tried. Healthcare professionals evaluated these approaches by sharing experiences with colleagues, fostering mutual learning.

### Rewarding yet challenging

Participation and learning contributed to several rewarding outcomes but also posed challenges, making it a balancing act to manage these; balancing between the group and the individual, between relatives and residents, between different professions, and between different interpretations of what participation meant. Healthcare professionals sometimes discussed possible solutions to the challenges together, but residents were rarely included in these conversations, their perspectives were seldom sought, and healthcare professionals had not even considered that possibility.

Rewarding outcomes of participation and learning included increased well-being, preservation of residents’ abilities, increased food intake, new knowledge, and greater job satisfaction. New insights were that some residents chose vegetarian dishes, which healthcare professionals had not anticipated, and that previously served portions were too large. Serving smaller portions, with the option to take more, reduced residents’ stress about not finishing their meals and wasting food.

However, participation and learning could also pose challenges that required active management. When participation was understood merely as complying with whatever the resident accepted or declined, it could lead to the risk of deteriorating health or suffering. Some healthcare professionals perceived that nurse assistants and licensed practical nurses at times lacked reflective practice, resulting in superficial knowledge. Openness to alternative approaches was limited, and reflection on how questions were posed or explanations provided were sometimes absent. In some situations, insufficient attention was given to clarifying why a particular action was important or the potential consequences of not doing it.

“Of course you should respect a no, but you can ask in different ways and do it in different ways…you may have to talk about something else, or go away for a while, and try again. Sometimes it’s easy to just, no, the person said no… maybe you put in extra effort to get it done if you know the background.” [Participant 10].

These challenges were perceived as linked to gaps in knowledge which could be improved through greater participation of registered nurses in the units, thereby fostering opportunities for mutual learning. The increased participation in the meal concept could pose challenges, particularly in units where residents had not previously participated in choosing meals and serving themselves. Some units encouraged greater participation despite residents’ initial resistance, whereas others prioritised residents’ immediate satisfaction and continued to serve them as before. Successfully increasing participation and learning was a balancing act, requiring encouragement at an appropriate pace to foster acceptance without causing distress. Once increased participation had been established, it was perceived as irreversible, and residents began to object if the opportunity was withdrawn.

Several challenges were related to tensions between the interests of the group and those of individual residents, where the participation and learning of different residents could come into conflict. For example, one resident’s participating and learning through eating independently could lead to deteriorating table manners, which other residents found disturbing. Participating through choosing where to sit could also be a challenge between individual autonomy and group considerations. Based on their knowledge of interpersonal dynamics, healthcare professionals sometimes avoided seating certain residents together, which limited residents’ opportunities to make their own choices.

Different forms of participation could conflict, creating challenges. For example, the possibility to choose dishes, combined with needs for texture modification and dietary restrictions, resulted in multiple separate canteens, which in turn led to a challenge to enable residents to serve themselves. Challenges also arose related to cognitive or physical impairment, such as uncertainty or confusion when presented with too many options, or insufficient nutritional intake when residents preferred to eat independently. Social participation was further challenged when residents wished to engage in conversations but were limited by hearing impairments, either their own or other’s.

The appropriate extent of relatives’ participation was perceived as challenging. Some healthcare professionals perceived them as valuable, generally wanting the best for their loved ones, and enhancing social interaction during mealtimes. Others perceived that relative’s presence could increase anxiety and reduce focus on eating among residents with cognitive impairment. Some healthcare professionals perceived that relatives lacked insight into the resident’s current situation, as they did not always recognise changes over time. Their outdated image of the resident could create challenges, such as requesting foods the resident could no longer eat or expecting participation in activities beyond their current abilities.

“They have difficulties seeing that mom is as sick as she is…mom has always eaten crispbread. But now it is not possible. It does not matter; she will have crispbread because she has always eaten it…knowledge is important to relatives, too…very important to understand the disease course…they have a wishful image of what mom has been like.” [Participant 8].

When relatives and residents expressed different preferences, healthcare professionals always prioritised the resident’s wishes and supported them in achieving their desired mealtimes experience.

Mutual learning between different professions could also be challenging, as registered nurses and licensed practical nurses worked under different managers and were governed by different legal frameworks. Although registered nurses were professionally responsible for care, they were not the managers of the licensed practical nurses who carried out the daily caregiving tasks. It was also challenging for registered nurses to provide education in nutrition without being fully familiar with the meal concept used within the units. Nevertheless, they expressed that they did their best to adapt the education to the needs of the group, which contributed to the possibility of mutual learning.

### Prerequisites influence opportunities

To enable participation and learning, prerequisites were necessary at multiple levels and when these conditions were absent, they acted as barriers. Healthcare professionals often found solutions and did their best under the circumstances, but the lack of appropriate conditions could lead to frustration. The theme includes three subthemes: prerequisites at the organisational level, the group level, and the individual level.

#### Prerequisites at the organisational level

At the organisational level, both structural and environmental prerequisites were essential. Structural elements to enabling participation and learning included having sufficient time for the meal, continuity among personnel, and adequate staffing. Insufficient staffing occasionally made social pedagogical meals impossible.

“We are not allowed…when we are feeding we are not supposed to eat (social pedagogical meal) at the same time due to hygiene (reasons), but, in one unit where you are alone during the meal, it is the only chance to eat together with them…you have to do two things at the same time.” [Participant 13].

Collaboration shifts were often experienced as stressful and fragmented, but they could also provide opportunities for mutual learning among colleagues. Scheduling was essential and poor organisation could become a barrier; when colleagues began their shift during meals, it could interrupt social participation. When activities were scheduled too close to mealtime, it often caused stress and reduced participation. Structures, such as the meal concept, were perceived as facilitating participation and learning, as the municipal decision to implement them across all units contributed to a shared goal. However, guidelines could also act as barriers; for example, hygiene-related restrictions prevented residents from being present in the kitchen.

Environmental prerequisites were also required, such as sufficient space in the dining room to ensure accessibility for residents using wheelchair or other assistive devices, and the possibility to adapt the furniture to enable residents to serve themselves and to facilitate participation for relatives. Tableware adapted for residents with physical or cognitive impairments was also perceived as important to enabling participation.

#### Prerequisites at the group level

The group climate and the capability to work as a team were important prerequisites at the group level. The notion of the group varied; most commonly it referred to colleagues, but in some cases, residents were also included. Relatives were less frequently included in this conceptualization. An atmosphere characterised by openness, tolerance, and support was important, as it enabled questions to be asked and openly recognised knowledge gaps.

“We all share our opinions, what we think, yes, and then it becomes easier to work…we have different suggestions and solutions, we always get to an agreement in the end, I think.” [Participant 15].

The group’s ability to organise work, distribute tasks, and adapt flexibly to the unit’s conditions were also viewed as prerequisites. To enable a social pedagogical meal for residents who chose to eat in their apartments, planning was required, sometimes resulting in meals being shared with these residents before or after the main mealtime. Adaptions needed to reflect both the competence and abilities of the healthcare professionals working a given shift, and the residents’ abilities and needs. Well-functioning teamwork required both structured communication, such as daily morning planning, and ongoing dialogue throughout the day. Preparation and planning contributed to a calmer mealtime situation, facilitating conversations and increasing the availability of licensed practical nurses to provide support, thereby enhancing residents’ participation and creating opportunities for learning. However, when agreed routines were not followed, opportunities for residents’ participation were reduced.

#### Prerequisites at the individual level

At the individual level, knowledge and a person’s view of humanity were important prerequisites. Required knowledge included factual and practical knowledge. Factual knowledge encompassed normal ageing as well as diseases and disabilities common in long-term care, and was essential for enabling residents’ participation tailored to individual circumstances. Knowledge of Swedish meal culture and sufficient language proficiency were also necessary to enable participation and mutual learning.

Familiarity with residents’ past and present habits, preferences, and wishes was a prerequisite. As some residents were unable to communicate verbally, knowledge of their non-verbal communication was crucial for fostering participation and learning. Practical knowledge entailed openness and responsiveness to rapid changes in residents’ conditions. However, knowledge about residents could also act as a barrier. Assumptions about a resident’s preferences could lead healthcare professionals to refrain from asking what the resident wanted, misinterpreting acceptance and satisfaction as a desire for nothing to change, and thereby missing opportunities for participation and learning.

Listening to residents, valuing their voices, and showing genuine interest in their views were regarded as essential components of a humanistic approach, grounded in a view of humanity that regards older adults as equal in value to everyone else. Such a view of humanity fostered an attitude oriented toward enabling residents’ participation and learning.

“That you see and hear the older, the elderly, that you see them as humans…sure, they are older, but they are not worthless. They have cognitive impairments, but they are not stupid, they are a human, a person who has lived.” [Participant 19].

However, there was occasionally a more reductionistic view of humanity, an attitude toward residents that placed greater focus on one’s own working environment rather than on what was best for the resident.

## Discussion

The result of this study showed that participation and learning related to mealtime situations can occur when appropriate prerequisites are met; however, managing the associated challenges appears to involve a constant balancing act. Healthcare professionals perceived learning from residents, their relatives, and colleagues as a natural and integral part of daily work. Nevertheless, learning was more often described in relation to colleagues than to residents and relatives. When residents and relatives are positioned as passive recipients rather than active participants, opportunities for mutual learning may be reduced. Learning as something implicit may reflect how healthcare professionals interpret learning, but it could also be related to a limited habit of reflecting on their practice and what influences it. Viewed through the lens of Illeris’ [41] learning model, it may be possible to identify more explicitly which parts of learning (content, incentive, and interaction) are being focused on, thereby enabling a deeper understanding of learning related to mealtime situations.

Among the healthcare professionals in this study, theoretical and practical knowledge were the most expressed content dimensions of learning. In relation to residents and relatives, the only learning content mentioned concerned behaviours and habits. In other studies [10, 52, 53] this is described as information rather than knowledge, which may depend on how knowledge is interpreted. According to Liedman [54], information can be transformed into knowledge when it is incorporated into one’s existing understanding, contextualised, and applied. Since the healthcare professionals in our study reflected on information about residents’ habits, and applied it in their interactions, this information can be understood as knowledge, thus forming part of the content dimension of learning. However, many residents and relatives possess extensive knowledge, experience, and insights in other areas as well. With greater openness from healthcare professionals, this knowledge could be utilised as a resource to address challenges in mealtime situations within long-term care facilities and thereby foster mutual learning.

The results showed that participation itself could be challenging, particularly when residents did not initially appreciate the change that participation in mealtime situations entailed. Previous research has reported both similar and contrasting findings, with some residents perceiving meal service as the responsibility of healthcare professionals [55], while others valued the opportunity to serve themselves and fellow residents [56]. In our study, some healthcare professionals prioritised residents’ immediate satisfaction by serving meals rather than enabling participation to the extent of their abilities. This may act as a barrier to residents’ learning by limiting opportunities to practise their skills, thereby negatively affecting the content dimension of learning and potentially leading to a decline in abilities.

A humanistic view of humans was an essential prerequisite for participation and learning in this study. In the incentive dimension of Illeris’ learning model, emotions, motivation, and volition are central components [41]. Healthcare professionals’ humanistic approach reflects both emotion and volition, thereby supporting learning and participation. Learning requires mental energy, mobilised through the incentive dimension [41]. For residents, this may be challenging, as they may lack sufficient mental energy for learning. In such situations, support from healthcare professionals may be appropriate. However, there is also a risk of underestimating residents’ motivation or energy, leading healthcare professionals to refrain from attempting to overcome the resistance that Liedman [54] describes as inherent to learning. A desire to protect residents and conserve their energy may reflect care and concern, but can unintentionally limit residents’ autonomy and opportunities to learn through preserving their abilities. This is consistent with Moilanen et al.’s [57] review, which found that healthcare professionals in long-term care emphasised protecting residents’ right to make their own decisions, but found it difficult to assess their capacity, often underestimating it. As a result, they tended to make decisions on residents’ behalf, reducing both participation and autonomy.

Sharing decision-making and participation are central to person-centred processes that enable persons to flourish. Flourishing may involve harmony and stillness, but also growth and to push boundaries [28]. When healthcare professionals in our study did not invite residents to participate, this may have reflected an intention to support flourishing through stillness and harmony; however, it may also have limited opportunities for growth by pushing boundaries. Expectations from others can encourage such growths, as feeling that others believe in one’s abilities may enhance motivation [58] and influence the incentive dimension, thereby enabling learning [41]. This relates to the humanistic view of humans, a prerequisite for participation and learning in our study, and to prerequisites for person-centredness such as self-knowledge and clarity of beliefs and values, both of which can be developed through individual and shared reflection [28]. Previous studies have shown the complexity of mealtimes and the multiple values involved [8], and our findings underscore the importance of awareness, along with sufficient time and appropriate tools, to work with these values to enable participation and learning.

The interpretation of participation may also pose challenges when it was understood simply as complying with whatever the resident accepted or declined. This reflects a superficial understanding, similar to the risk of applying person-centredness in a superficial way [59], interpreted as a passive process, where healthcare professionals simply respond to what a resident explicitly expresses. However, according to the Person-centred Practice Framework [60], healthcare professionals need the ability to perceive nuances, the signs of the resident, to enable shared decision-making and engage authentically with them. Otherwise, there is a risk that healthcare professionals assume responsibility for addressing a resident’s needs without enabling their participation. Thus, healthcare professionals must be able to step back to create space for residents to act in accordance with their own convictions, while simultaneously being prepared and confident to intervene on their behalf when residents are in a vulnerable state. Achieving this level of knowledge and understanding requires time for reflection. A possible approach is to use narrative portraits, where narratives from residents, collected through interviews, are used for reflection and discussion among healthcare professionals. This has been shown to support both individual and collective learning, and to improve the quality of care [61].

In our study, healthcare professionals interpreted residents’ silence as an indication of satisfaction with the mealtime situation. However, previous research showed that silence among older adults in long-term care facilities may serve as an expression of loyalty toward healthcare professionals, aimed at not burdening them, or as an expression of the realisation that they are unable to influence their situation, leading them to lower their expectations and adapt to the situation [62, 63]. Silence has also been described in the review by Harstäde et al. [55], in which residents, despite wanting more food, refrained from asking when they perceived healthcare professionals as busy. This underscores the importance of education and reflection among healthcare professionals to move beyond the surface-level, to interpret and better understand what silence and other behaviours may represent. Curiosity about and engagement with residents are essential to achieving this. One way to foster such an approach may be through deeper implementation of person centredness, in which these values are fundamental [60]. Such an approach may also help reduce variation in residents’ opportunities for participation found among healthcare professionals in our study, thereby enhancing learning opportunities.

Regarding relatives, their motivation and volition were perceived by healthcare professionals as positive to a certain extent, but beyond this limit, they were sometimes perceived as troublesome. This aligns with previous research, describing the dual perception of relatives as both valuable contributors and troublemakers [36, 64]. By creating opportunities, allocating time, and providing appropriate settings for collaboration, barriers between healthcare professionals and relatives may be reduced, thereby enabling mutual learning.

Among healthcare professionals in our study, several forms of interaction occurred, such as sharing experiences, imitation, and participation. These can be linked to the interaction dimension of the learning model, which consists of communication, cooperation, and action [41]. However, lack of knowledge and insufficient language skills among healthcare professionals challenged interaction and could act as barriers to learning. Healthcare professionals also appeared to determine the extent to which residents and their relatives were allowed to interact with them. Residents were often invited into more passive forms of interaction, such as observing and receiving, rather than actively participating in addressing challenges in mealtime situations. Within the interaction dimension, sociability develops [41], and may be both natural and important for healthcare professionals. However, residents in long-term care facilities may also be compelled into certain forms of sociability, to adapt to their required form of living. This reflects one of the challenges identified in our study, i.e. the tension between individual and group interests. Routines in long-term care facilities are often designed to meet the needs of many, which can make it difficult to address individual needs [65]. Similar challenges have been described in previous research, for example when residents had to be excluded from shared meals due to conflicts with other residents [8], and when group needs are prioritised, residents may experience reduced autonomy [66], thereby limiting opportunities for participation and learning. Although mealtime situations in long-term care facilities are complex and no single solution fits all, improvement may be achieved by involving residents and relatives more actively in problem-solving, and by increasing reflexivity among all those involved. However, to achieve this, further research on residents’ and relatives’ perceptions of participation and learning is needed.

### Methodological considerations

The sampling aimed to achieve variation in characteristics among participants, and regarding age and experience, heterogeneity was achieved. However, all nurse assistants and licensed practical nurses were or had been mealtime representatives, which may be considered a limitation for transferability. On the other hand, their greater knowledge of mealtimes in long-term care facilities may have contributed to a deeper understanding of participation and learning related to mealtime situations as they may have been better able to perceive and describe the phenomena. The study was conducted in one region, which may be seen as a limitation; however, it included four long-term care facilities located in both central and rural areas, which can be considered to strengthen transferability. We believe there is conceptual coherence between theoretical assumptions, research question, design, and methods, which may strengthen trustworthiness [43]. The first author had no prior relationship with the participants and no experience working in the study context. However, previous experience in pedagogy and supervision may have contributed to a deeper understanding of the data during the analysis. Within the research group, there is knowledge of both mealtime situations and pedagogy, which through awareness, discussion, and reflexivity may have contributed to the confirmability of the study. Documented reflections, the reflexive journaling by the first author, and the illustration of the themes through data extracts from across the dataset, including extracts from different participants, could also be considered valuable for confirmability.

## Conclusions

Participation and learning among residents, their relatives, and healthcare professionals related to mealtime situations are complex processes, requiring active efforts at all levels of the organisation. These efforts can foster participation, which in turn may create opportunities for learning and knowledge exchange. Healthcare professionals balance multiple challenges and strive to act in residents’ best interests; however, residents and their relatives are seldom involved in how these challenges are addressed. Deepening person-centredness in long-term care facilities may create opportunities for participation and learning to one’s desired level.

By applying the dimensions of learning through the lens of Illeris’ learning model, the complexity of learning in different mealtime situations became more visible. To further support the development of learning-oriented long-term care practice involving residents, relatives, and healthcare professionals, our results indicate that such an approach may be beneficial.

## Supplementary Information

Supplementary Material 1.

## Data Availability

The datasets analysed during the current study are not publicly available as it is difficult to fully anonymise qualitative data, but data are available in Swedish from the corresponding author on reasonable request.
