# Supplementary material for: Healthcare professionals’ perceptions of participation and learning related to mealtimes in long-term care facilities: an interview study
Source: BMC Geriatr. 2026 Sep 24;26:1212. doi: 10.1186/s12877-026-08358-x (PMC13615625; doi:10.1186/s12877-026-08358-x)
Supplement: Supplementary file 1 [file 12877_2026_8358_MOESM1_ESM.docx]

**Supplementary file 1. Interview guide**

Mealtime situations are a recurring part of daily life in long-term care facilities. What do you think is important for a good mealtime situation?

If you imagine a typical mealtime situation for the residents at your workplace, who participates in it?

In what way are they allowed to participate?

Follow-up questions addressing key aspects of mealtime situations, such as the meeting, the room or location, the food and beverages, and the organisation.

When people participate, it can also mean an opportunity to get new ideas and learn new things. How do you see the opportunity to learn from and with colleagues, residents, and relatives?

Probing questions

Can you explain?

Can you give an example?

How do you handle suggestions?

In what ways could you learn?
